# “Invalid busyness” behaviors of a few grassroots cadres: Evidence from normative explanation

**DOI:** 10.3389/fpsyg.2022.1027427

**Published:** 2022-11-23

**Authors:** Cheche Duan, Yan Zeng, Yicheng Zhou, Yingying Li

**Affiliations:** ^1^School of Government, Shenzhen University, Shenzhen, China; ^2^Excellent Innovation Team of Local Government and Social Governance, Soochow University, Suzhou, China; ^3^Shenzhen Airport, Shenzhen, China

**Keywords:** grassroots cadres, Invalid Busyness, cadre incentive, grassroots governance, taking-charge behaviors

## Abstract

Effective grassroots governance is the foundation for the stability of state power, but the “invalid busyness” behavior of a few grassroots cadres has seriously deteriorated the local political ecology. In order to gain a deeper understanding of the mechanism of “invalid busyness” behavior of grassroots cadres, this paper, following the analysis of typological research and combined with publicly available data, classifies the “invalid busyness” behavior of grassroots cadres into eight types, including hedging and self-preservation, drifting with the stream, patchwork response, conforming old rulers, replacing targets, blame avoidance, trace doctrine, and self-waiver. It is found that the “invalid busyness” behavior of grassroots cadres is shaped by the risk society paradox, local management paradox, incentive intensity paradox and technical governance paradox, and the “invalid busyness” behavior of grassroots cadres needs to be corrected from the four-dimensional logic of incentive, restraint, deep care, and strict control. This study systematically describes the manifestation, causes and correction mechanism of the “invalid busyness” behavior of grassroots cadres, which enriches and expands the theoretical research on the performance behavior of grassroots cadres.

**Systematic review registration:**

https://osf.io/h5wgj.

## Introduction

With the rise of the New Public Management (NPM) movement in the 1970s, the issue of “bureaucracy disease” has increasingly come to the forefront of academia and has become an important topic in public management research (Bozeman, 2000; Ma, 2010). The new form of the bureaucratic disease is the “invalid busyness” of public officials, which is mainly reflected in “red tape,” “traceism,” and “formalism” (Merton, 1940; Bozeman, 2000; Yan and Yang, 2019). Not only does it reduce administrative efficiency, but it can also inhibit the work dynamics of public officials, which has a negative impact on the development of public service motivation (Moynihan and Pandey, 2007).

In fact, the “invalid busyness” behavior of public officials not only consumes a lot of resources and energy, but also is a persistent problem in national governance. The Oxford Dictionary defines red tape as “unnecessary and cumbersome rules and regulations, which usually lead to delays and difficulties” (Ma, 2010). Peyrefitte (2006), a French politician, pointed out the “French disease” in his book *Le Mal Françai* that permeates the political system which uses records, reports, studies, statements, and appraisals to create a false impression of a comprehensive grasp of the situation, only makes statistics but neglects the living reality, and does what should not be done rather than what should be done. The campaign of “remodeling the government” which began in the early days of former US President Clinton's administration aims to cut red tape in the bureaucratic system to make the government more efficient (Gore, 1993).

Busy is the performance of officers and the basis for success. There is nothing wrong with being busy, especially after the reform of the New Public Management Movement, as the grassroots government and its staff, who are the link between the state and the society, have taken on more affairs and become busy as a norm, but there is a need to prevent invalid busyness and blind busyness. For this reason, the bureaucracy has taken a series of measures to remedy the problem of “invalid busyness” in grassroots governance, such as strengthening the training of public service motivation, strict politicized recruitment mechanisms for bureaucrats, and expanding the use of modern information technology (Peters and Pierre, 2004: 2; Welch et al., 2005; Homberg et al., 2019), in order to free the hands and feet of cadres from “invalid busyness” affairs and motivate them to be busy acting and doing real work. However, the grassroots seems to be caught in a strange circle of “the more the burden is reduced,” and “invalid busyness” is growing and spreading in grassroots governance, even evolving into a kind of unorganized collective action, which seriously affects the overall morale and motivation of cadres, and also affects the smooth promotion of the national cause (Zhou, 1993; Yang, 2022). Why do grassroots cadres fall into “invalid busyness” even though they know it is ineffective? What corrective mechanisms can effectively reverse the trend of “invalid busyness”? To this end, this study focuses on a hierarchical analysis of the phenomenon, root causes, and generation mechanisms of “invalid busyness” of grassroots cadres and explores the theoretical basis and practical reference for solving the problem of “invalid busyness.”

## Research background and literature review

The performance and style of grassroots cadres have always been important research content of grassroots governance, especially in recent years, with the shift of the center of gravity of public governance and the reform of “management and service,” many scholars have paid attention to the behavioral changes of grassroots cadres. However, the academic research on the “invalid busyness” behavior of grassroots cadres is still relatively fragmented, and the theoretical discussion is mainly carried out from the following aspects.

### The manifestation of “invalid busyness”

The busy status of grassroots cadres is a concrete manifestation of performing their duties and responsibilities, but excessive busyness can also bring negative effects to grassroots governance. Most of the existing studies measure the “invalid busyness” behavior of grassroots cadres by the following two indicators: from the perspective of work quantity, in addition to regular administrative affairs, grassroots governments have taken up a large number of “veto” temporary affairs in recent years, such as, “Day+Night,” “5 + 2” and “7 × 24” have become the norm for grassroots cadres (Tu and Gong, 2021; Yan and Yang, 2022); from the perspective of work quality, grassroots cadres spend a lot of energy in filling out forms, reporting, meeting inspections and receiving assessments, and their time to serve the public is thus squeezed (Tummers et al., 2015; Gao, 2017). These two indicators are mutually influential, working too long hours tends to make people burned out, and poor performance cuts down on public employees' self-efficacy, which in turn can reinforce public employees' burnout and weaken their motivation to serve the public (Wen and Zhang, 2017; Lu and Guy, 2019). In addition to the above studies, some scholars have also summarized the manifestations of “invalid busyness” behavior. For example, Yang and Li (2020) argue that “busy but useless” is a concrete manifestation of traceism and involutional governance, which means that a lot of time and energy are invested without achieving corresponding results, but instead solidify the contradictions of grassroots governance; Chen (2020) describes the busy behavior of grassroots governance, in which everyone handles and leaves traces everywhere but does not help to solve problems, as “partial idling;” Tu and Gong (2021) confirm through a questionnaire that diverse and even conflicting behavioral requirements can squeeze the intrinsic motivation of grassroots cadres and weaken their responsiveness to the public.

### Causes of “invalid busy”

The “invalid busyness” behavior of government officials, like many other social phenomena, has multiple causal mechanisms, and is analyzed mainly in the following three aspects. First, the pressure of the hierarchical structure. Pressure-based institutions are an important concept in understanding the operation of hierarchical institutions, emphasizing the state of government at all levels driven by various pressures (Yang, 2012). However, existing studies have found that as section-level pressures continue to intensify, some government officials have emerged with explicit or implicit motives to blame accountability (Norman, 2002; Ni and Wang, 2017). In particular, grassroots officials, who are at the interface between the state and society, bear extremely heavy governance tasks, and formalistic “invalid busyness” behavior becomes a rational behavior to avoid accountability risks (Tu and Gong, 2021). Second, the institutional design is flawed. New institutionalism believes that institutions are the most solid method of governance, but the lack of institutional effectiveness is also an important cause of governance problems (North, 1990). On the one hand, deficient institutional design can lead to the unclear division of labor, resulting in the transfer of a large number of tasks to lower levels of government and exacerbating the “invalid busyness” behavior of grassroots governance (Lieberthal, 1992; Sminth, 2010). On the other hand, the lack of adaptability of institutional design can also lead to “invalid busyness” behavior, because the modern state has also experienced the transformation from modernization to modernity in the process of governance, also facing the problem of “new system is not enough, the old system does not work,” and then the alienation behavior that is contrary to the original design of the system (Huntington, 1968; Gao, 2017). Third, there is a lack of political responsibility. From a public person's perspective, government officials usually have a higher motivation and dedication to public service than the staff of social organizations, and are engaged in doing “things that can make society better” (Bovens, 2010; Perry et al., 2010; Zhang and Li, 2018; Lyu, 2020). From a societal perspective, government officials are also rational economic agents who seek to maximize their personal interests and seek to minimize or not to lose their own interests when the public interest conflicts with their personal interests (Suchman, 1995; O'Brien and Li, 1999). Among them, the “invalid busyness” behavior is a strategic compromise made by the government based on the trade-off between the dual roles of “public person” and “social person” (Yang and Li, 2020).

### Governance of “invalid busyness”

In contrast to the manifestation and causes of the “invalid busyness” behavior, the governance mechanism of the “invalid busyness” behavior is also an important part of scholars' research, there are three main views. First, it is advocated that the autonomy incentive of grassroots cadres should be enhanced. Unlike the previous economic and promotion incentives, the autonomy incentive refers to the belief that grassroots cadres have the ability to solve grassroots affairs, so that grassroots cadres can adjust their governance behavior according to the actual governance scenarios in order to enhance their self-efficacy (Thomann et al., 2018; Liu and Xu, 2021; Ou and Wang, 2022). Second, more resources should be sunk to the grassroots. With the modernization of grassroots approaches and the increasing demands of the people, grassroots governments are taking on more and more governance functions and political responsibilities, with the tendency of administrativeization (Hou, 2019). Therefore, it is necessary to match the grassroots government with corresponding human, material and financial resources, etc., in order to enhance the working ability and effectiveness of grassroots cadres (Pfeffer and Salancik, 1978:29; Yang and Yu, 2012; Tummers, 2016). Third, cutting out the formalism in grassroots governance. Taking people's satisfaction as an important index for grassroots cadres' assessment, cut out unnecessary paperwork and traceism in order to release grassroots cadres from all kinds of formalism, so as to motivate grassroots cadres to do real work and realize the unity of productive and effective government (Karl, 1940: 51–53; Duan, 2021; Jiang and Wu, 2021).

In general, the existing studies have analyzed the “invalid busyness” behavior of grassroots cadres from different perspectives and directions, which provide a theoretical reference for this study. However, there are also some shortcomings. First, the existing studies on the “invalid busyness” behavior of grassroots cadres are still fragmented and have not yet clarified the basic connotation of the “invalid busyness” behavior, and lack a systematic condensation and overview of the “invalid busyness” behavior of grassroots cadres; Second, most of the existing studies have theoretically explained the taking-charge behavior of grassroots cadres from a single dimension, but still lack of an overall framework to clarify the logic of the generation of “invalid busyness” behaviors of grassroots cadres; Third, the existing research provides general policy suggestions for the correction of alienated behaviors of grassroots cadres, and it is still necessary to provide targeted correction measures for the “invalid busyness” behaviors of grassroots cadres in combination with their typical performance and formation mechanism. In view of this, based on the existing theoretical research and practical materials, this paper conceptualizes and classifies the “invalid busyness” behaviors of grassroots cadres, explores the generation mechanism of “invalid busyness” behaviors under the theoretical framework of “environment, organization, institution, technology, and political man,” and puts forward the correction measures for the “invalid busyness” behaviors of grassroots cadres from the four-dimensional logic of “incentive, restraint, deep love, and strict control.”

## Conceptual deconstruction and types of “invalid busyness”

Based on the existing research, this part deconstructs the concept of grassroots cadres' “invalid busyness” and tries to systematically sort out the typical performance of grassroots cadres' “invalid busyness” by combining the in-depth reports of media such as *China Comment, Xinhuanet* and *National Governance*.

### Research method

Typology, also known as “taxonomy,” is a system of grouping and categorization, essentially an analytic and inductive epistemology, whose role is to provide the basis for deeper research. As Mill (1860) says, “The universe as we know it is so constituted that the truths that exist in any event are true in all cases of a certain kind; the only difficulty is to discover what kind.” The comprehensive and correlative characteristics of the typological research method can comprehensively extract the information contained in the research object and summarize the common characteristics of the research object in order to deepen the knowledge and understanding of the research object. So far, the typological research method has been widely used in the fields of sociology, anthropology and political science, and has strong theoretical adaptability.

### Basic connotation of “invalid busyness”

“Invalid busyness” refers to the manifestation of the involution of grassroots governance in the behaviors of the governance entities (cadres), “busyness” refers to the input and consumption of administrative resources, and “invalid” refers to the shelving of services and avoidance of contradictions in administrative work.

The concept of involution originated from the regressive theory of Kant (1790), matured in the Cultural involution of Goldenweiser (1936) and the agricultural involution of Geertz (1963), and then expanded to anthropology, economics, sociology, administration, and politics. Involution extends to the collective, dissipative, and repetitive organizational pathological behaviors of the cadre group, reflected in the “invalid busyness” behaviors of the grassroots cadres, that is, while the state inputs more, the grassroots cadres become all the busier, but the efficiency is getting lower. The “invalid busyness” of grassroots cadres refers to the fact that, under strict external restrictions and multiple internal constraints, grassroots cadres have invested plenty of time and energy and consumed a lot of administrative resources in the process of handling administrative affairs, consciously or unconsciously indulged in refined, complicated and technical administrative forms, administrative links and administrative processes, but failed to produce the desired results, and fell into a state of collective self-dissipation and governance suspension.

From the perspective of motivations, the “invalid busyness” behavior of grassroots cadres is a stress response to changes in the internal and external environment of the organization. Especially with the significant increase in the difficulty of reform and innovation and the pressure of supervision and discipline enforcement in recent years, the grassroots cadres, as rational economic persons, are more inclined to seek self-protection in moderate compliance and cautious resistance (Williamson, 1987).

From the perspective of orientation, the “invalid busyness” behavior of grassroots cadres is a typical manifestation of emphasizing “process orientation” but ignoring “result orientation.” The heterogeneity of results is covered up by the legitimacy of procedures. Moreover, such “invalid busyness” behaviors will also have an infectious effect, resulting in irrational behavior of “action without effect” in grassroots governance.

From the perspective of behavior impact, the “invalid busyness” behaviors of grassroots cadres will not only delay favorable opportunities for the development of the Party and the state, but also weaken the efficiency and level of grassroots cadres' response to the demands of the people, and make the grassroots cadres spend limited time and energy on “invalid busyness” affairs such as having meetings, dealing with supervision, statistics and tabulation, which is not conducive to the modernization of grassroots governance system and governance capacity.

### Types of “invalid busyness” behaviors of grassroots cadres

Studies have focused on measuring the “invalid busyness” behavior of grassroots cadres in terms of “work quantity“ and “work quality,” but neglected the fact that grassroots cadres, as dynamic individuals, will adjust their behavior according to the changes in system, environment, and structure, etc. Therefore, in order to analyze the “invalid busyness” behavior of grassroots cadres more precisely, we can adopt the “practical awareness” and “behavioral competence” according to Giddens' structural functionalism.

“Practice consciousness” is the basis of Giddens' structural functionalism, which dissolves the dichotomy between action and structure and provides enlightening insights for understanding the “invalid busyness” behavior of grassroots cadres (Giddens, 1986: 6). On the one hand, low behavioral capacity-passive practice awareness: conforming old ruler/trace doctrine. The “invalid busyness” behavior is the passive response of grass-roots cadres in the face of multiple task requirements, which is mainly reflected in their own incompetence and improper pressure transmission and passive abandonment of professional autonomy, which inevitably results in the loss of individual autonomy of cadres and mechanical repetition of coping with work, leading to the alienation of trace management into traceism. On the other hand, high behavioral capacity-proactive practice awareness: self-preservation/blame avoidance. Facing the increasingly heavy pressure of regulation and accountability, the political consciousness of some grassroots cadres gradually alienates into self-preservation of responsibility avoidance, which is mainly reflected in the fact that some grassroots cadres are busy coping with the assessment and supervision of higher levels, and even jointly coping with the assessment and supervision of higher administrative bodies to avoid accountability and responsibility.

Many things are not my intention to do, or not what I want to do, but are caused by me, no matter how, I did do (Giddens, 1986:8). The “do” here refers to the behavioral ability possessed by the actor, and thus distinguishes the “invalid busyness” intention and behavior of grassroots cadres. On the one hand, low behavioral capacity—proactive practice awareness: drifting with the stream/self-waiver. Under the double pressure of performance target and responsibility risk, grassroots cadres as rational individuals prefer to choose those work areas with low risk, small investment and quick results, which leads to the state of self-loafing, self-consumption, and passive coping of grassroots governance and cadres. The “optimal solution” for some grassroots cadres to avoid responsibility. On the other hand, high behavioral ability—passive practice awareness: patchwork response/replacing target. In the process of building various e-government platforms in full swing, due to the lack of a collaborative data sharing mechanism and interoperability platform, grassroots cadres often need to duplicate and report data and reports to different business departments, which increases the workload of grassroots cadres and makes some tasks difficult to complete only by passively coping with or replacing governance goals.

**Figure 1:**
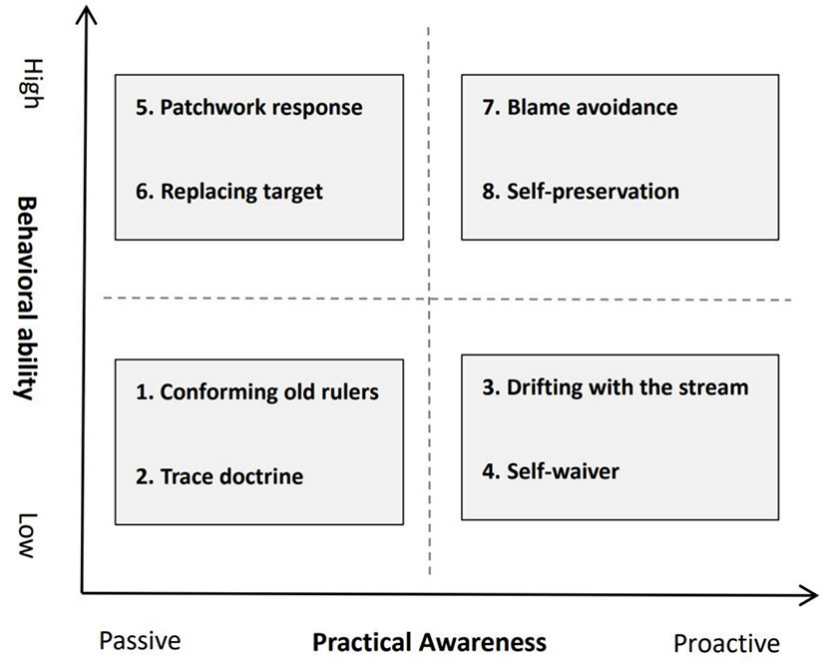
Classification criteria of “invalid busyness” in grassroots cadres.

The above two dimensions together constitute the basis and categorization criteria for analyzing the “invalid busyness” behavior of grassroots cadres in this paper (as shown in Figure 1), and the specific performance of the “invalid busyness” behavior of grassroots cadres will be analyzed one by one in the following.

#### Conforming old rulers

Some cadres with long credentials or elder age have rich experience in work related to the public, but they are obviously conservative about new knowledge, new technology and new concepts, unwilling to change their thinking mode and working methods, and seldom take the initiative to participate in skill training programs, complicating problems, and expanding complex problems in their work. Although the number of cadres who follow the rules and are old-fashioned and “busy for nothing” is not large, the negative effect is great, and it is easy to undermine the morale and atmosphere of grassroots cadres to build a career or a business.

*Some old cadres often “don't know how to operate a computer” as a reason to shirk their share of work, even if there is an opportunity to train computer knowledge, some old cadres are not active. It is worth being alert to the fact that the number of old cadres who seek no merit or demerit in the unit is small, but the impact is great and can easily bring down the entire unit's ethos. (BYT-20211026)*

#### Trace doctrine

The original intention of trace management is to make things that cadres do traceable and verifiable by means of making notes, taking photos and recording videos. However, when some leading cadres mistakenly regard “traces” as political achievements and ignore the fact that the grassroots work is heavy, extensive, with heavy tasks and many variables, the trace doctrine that deviates from reality to reality not only occupies a lot of time and energy of grassroots cadres, but also consumes a lot of public resources. However, it is difficult to implement the principles and policies of the Party and the state.

*A grassroots cadre said to the reporter: “At one time, the higher level required the production of files, a poor household a file of 24 pages, four copies of a total of 96 pages, but also photos, all files are packed with plastic leather. 158 households in the village, using 15,000 sheets of A4 paper, photo printing is unusually expensive ink, the toner cartridge used 13”. (XHW-20180816)*

#### Drifting with the stream

Grassroots cadres are not only “governance bureaucrats” at the junction of national governance and social governance, but also “street bureaucrats” who are closest to and most familiar with the people. “Busyness” is not wrong in itself, and overtime is inevitable. Purposeful and effective busyness are a manifestation of cadres' responsibilities. However, some cadres seem to go to work in advance and work overtime every day, but in fact they are just like drifting with the tide. They have a reputation for diligence but with no achievements. They use tactical busyness to cover up laziness and extravagance and seek self-consolation by “hard work even though with no credit.”

*The uncontrolled working style of 5*+*2, day*+*night is the culprit that gives rise to the feeling of anxiety. However, there are some places and individual units that still regard unstructured overtime as the norm and take unstructured overtime as a sign of dedication, and a very few people even gloat about taking the credit for eating and sleeping in the office all year round as the year-end summary. (XHW-20180523)*

#### Self-waiver

The “invalid busyness” is reflected in the performance behavior and work attitude of two types of cadres. First, some cadres who have encountered the “ceiling” of promotion see no hope of promotion and no “bright” future. They do not express their attitude and keep quiet when they encounter problems and are busy with forms and trivial matters all day to cover up their job burnout; Second, some cadres with weak public service motivation are content with the status quo and work with the mentality of “being the monk for a day, striking the bell for a day.” They seem to be busy, but they are playing idle work, and have become truly “Buddha-like” cadres.

*A grassroots cadres who have participated in the work of poverty alleviation in the village said that some long-term in the township front line, experienced, and rich middle-aged cadres, should have been the backbone of the township work, but due to overage promotion is hopeless, work enthusiasm gradually faded. At the same time, the reporter also found in the research, some units to find the office director, workstation station chief candidates are not easy to find, “sometimes also rely on favors, departmental leaders to talk in advance, do through the ideological work, they are willing to come.” (BYT-20211223)*

#### Patchwork response

“Insufficient work is made up with materials” is another epitome of the “invalid busyness” behavior of grassroots cadres. Work reports, leaders' speeches, research reports and other written materials are effective carriers for promoting and implementing the work, and important supports for testing the implementation of the work. However, in the face of some tasks that are time-consuming and urgent, grassroots cadres have no time to fulfill them but only to work overtime and rack their brains to “create” materials to piece together the materials to cope with the inspection and supervision of the superiors. Such a scenario is by no means a single case in grassroots governance. It not only makes grassroots cadres feel miserable, but also is not conducive to solving the “urgent difficulties and worries” of the people.

*A traffic management department cadres said: “In the writing of the local traffic accident handling materials, the superiors only asked to report the amount of violations, accidents and other sets of data, but unit leaders think it is too simple to write, and asked to add how the leadership attached importance, held several meetings, made several instructions and other content. In this way, the original 1 page can say clearly, was expanded to 6 pages. As a result, after the material was submitted, the higher authorities only extracted a few sets of key figures, and the effort to write things did not come in handy.” (BYT-20180926)*

#### Replacing targets

In recent years, as the focus of governance has sunk to the grassroots level, it has become normal for grassroots cadres to work from morning to night and from Monday to weekend. While it is still difficult to complete the “prescribed actions,” some grassroots cadres who have dual roles of agent and social person, on the one hand, make use of the asymmetry of information and the gaps in institutional designs to discount, make choices and make adjustments in the process of fulfilling the task; On the other hand, in the context of the reform to streamline administration, delegate power and improve regulation and services, the functions, authorities and affairs of higher-level departments have gradually moved down to the grassroots level, but they have neglected the more essential issues such as how to make the grassroots fulfill the tasks efficiently and effectively. In the face of the increasing task pressure in a short time, some grassroots cadres have no choice but to replace their targets or resort to fraud.

*A reporter learned: “a city in central this year to combat yellow-labeled vehicles (high pollution emission vehicles), this would have been a great thing, but the superior documents one-sided emphasis on cleaning up thoroughly, or performance assessment ranking points deducted, resulting in a “fake governance” farce staged. In order to complete the task, the traffic police team directly in the system first cancels the vehicle, but a large number of yellow-labeled cars actually still running on the road, and safety hazards have not been eliminated; some vehicles are not to cancel the scrapping time, but the superior “one size fits all” requirements to eliminate, the lower level had to take a sum of money to compensate owners. (BYT-20181015)*

#### Blame avoidance

Under the joint effect of the “responsibility and benefit” mechanism, the grassroots government and the direct superior government form an interest community. To cope with the regular inspection and target acceptance from the higher-level government, the direct superior government has collusive motives and tendencies toward the grassroots government (Zhou, 2010). In fact, the direct superior government or functional departments also know that the behavior of shirking responsibility to the grassroots cannot fundamentally shirk their own leadership and supervision responsibilities, and they will also be punished if there is a problem. Therefore, there is “invalid busyness” for blame avoidance, and “dealing with formalism with formalism” has become a tacit understanding between the upper and lower governments.

*In fact, the direct superior government also understands the actual situation of the grassroots government, and knows that some tasks are not really implemented at the grassroots level, but in the context of “increase in layers”, can only “turn a blind eye”, in some non-critical issues and pass by. To ensure that the grassroots government in the normal operation of the overload state but not to collapse. (GJZL-20210207)*

#### Hedging and self-preservation

Under the dual pressure of fuzzy governance and strict accountability, faced with the ever-increasing work tasks and responsibility requirements, some cadres only focus on doing things, fail to distinguish the priorities and grasp the key links, and then adopt the coping strategy of “putting quantity before quality,” and cover up the strategic confusion with tactical busyness; In addition, under the background of strictly administering the Party in an all-round way, “accountability” has become a sharp sword hanging over the heads of cadres. Some leading cadres even take accountability as a tool for daily supervision and work implementation, which causes some grassroots cadres to deal with it mechanically in order to reduce or avoid potential accountability risks.

*A cadre in a central county said: “the current poverty alleviation, environmental protection and other heavy workloads, such as true negligence, slack inaction, accountability is necessary, but there is some serious, hard work is still accountable, inevitably resulting in grass-roots cadres do not dare to act, unwilling to act, with ‘empty busy’ to cope with the work of situation”. (BYT-20181015)*

## Generation mechanism of “invalid busyness”

**Figure 2:**
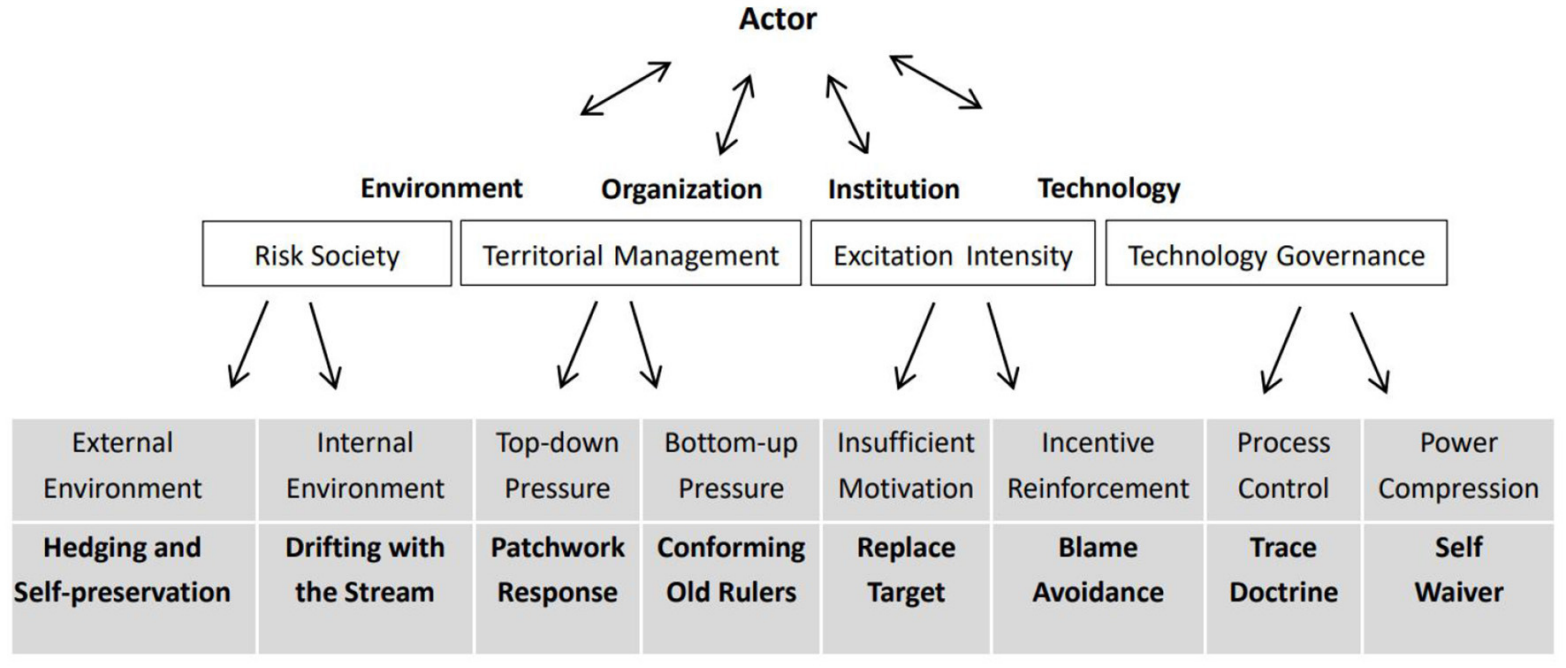
Types and causes of “invalid busyness” in grassroots cadres.

Like other social phenomena, the “invalid busyness” behavior of grassroots cadres has multiple inducing and generating mechanisms. It is affected by multiple factors such as mechanism design, environmental changes and organizational structure, which jointly shape the “invalid busyness” behaviors of grassroots cadres. Therefore, according to the basic framework of “environment, organization, institution, technology and political man,” this study explores the multiple game logic including environment (risk system), organization (power and liabilities system), institution (incentive system), technology (process system), and political man (cognitive system) (as shown in Figure 2) to explain the process of individual value judgment and behavior selection of the “invalid busyness” of grassroots cadres.

### Risk society paradox: Actor-environment

The environment has a subtle influence on the behavior selection of actors. It can better explain the internal mechanism that drives the behavior of grassroots cadres to take account of the “invalid busyness” behavior in the context of the environment where the organization is involved. At the macro level, the transformation trap of “compressed modernization” and the rise of civil rights awareness in China require cadres to take the initiative to act. However, the mismatch between the supply of the governance system and the demand for the governance environment is a serious bottleneck that restricts the improvement of grassroots governance capacity and causes cadres to deal with the pressure of the governance environment with “invalid busyness.”

At the micro level, in recent years, the internal and external environment of government organizations is also undergoing subtle changes. From the perspective of the external environment of the organization, social development leads to an unlimited demand for government responsibility. When the unlimited expansion of government responsibility makes them have too many problems to tackle, the actual responsibilities that government officials need to bear are often greater than their written responsibilities. Moreover, the “risk society” predicted by Ulrich Beck has become a reality (Beck, 1992). Natural risks and human risks, internal risks and external risks are intertwined. Facing the double pressure of unlimited responsibility and risk society, cadres have the opportunistic tendency of “invalid busyness.”

From the perspective of the internal environment of the governmental organizations, although the pragmatic sector has issued the notice and guidance on reducing the burden of the grassroots in recent years, a large number of political and administrative affairs can still sink to the grassroots through a new form, supplemented by the strong accountability measure of “one-vote veto,” which makes the grassroots cadres busy “benchmarking” the assessment indicators of the superiors and “implementing” the apportionment tasks of the superiors. Accordingly, some cadres have even fallen into the cognitive misunderstanding that “whether being busy or not is a matter of attitude, and whether achievements can be made after being busy is a matter of ability.” In the long run, this is not conducive to the improvement of grassroots governance capacity, moreover, it may lead to greater governance crisis and risk.

### Territorial management paradox: Actor-organization

A top-down power structure of governments is adopted in China, including governments at “central, provincial, municipal, county and township” levels, which corresponds to the bottom-up responsibility system. The principle of territorial management aims to effectively bridge the governance gap and enable governments at all levels and functional departments to assume the governance responsibilities within the territory. However, in the actual political scene, the enhancement of territorial responsibilities at all levels is alienated into a tool to shirk responsibilities at all levels, which makes the grassroots fall into the dilemma of “small power but big responsibility, many affairs but scarce resources and insufficient capacity,” unable to conduct effective management.

On the one hand, there exist serious power and responsibility mismatches between the upper and lower levels of the hierarchical organization and the upper and lower ranks of the cadres. In particular, the grassroots governments and cadres at the bottom of the power pyramid actively or passively undertake a large number of responsibilities transferred from the higher-level governments and functional departments. Moreover, the functional departments at the upper end of the power chain can also use their own power advantages to strengthen the responsibilities of the local grassroots governments. Faced with the constraints of multiple task requirements and “fragmentation” of resources, grassroots cadres at the bottom of the hierarchy must spend a lot of time and energy dealing with a variety of examinations, inspections, evaluations, etc., and grassroots governance has fallen into an “invalid busyness” state of self-consumption and passive response.

In the new era, the main social contradiction in our country has shifted to the contradiction between the people's growing needs for a better life and the unbalanced and inadequate development, which requires the territorial government to respond to the new needs and expectations of the people with a more responsible attitude. However, some grassroots cadres in the “sandwich layer” of power and the governance transition period, facing the ever-changing governance environment, have fallen into the situation of outmoded administrative concepts, outdated knowledge structure and weakened ability to perform duties. Moreover, some organizations still have serious bureaucratic, formalistic, and other bad work styles, which coerce individual actors in the organization, and try to cover up the embarrassment of insufficient ability by means of following the rules in an “invalid busyness” manner.

### Incentive intensity paradox: Actor-institution

In an ideal state, institution is a kind of game rules, aiming to reduce the uncertainty of people's behavior (North, 1990) and to provide behavior rules and expectations for the performance of grassroots cadres. As a result of the acceleration of the rule of law and institutionalization in China in recent years, a large number of informal or semi-formal rules have gradually withdrawn, however, the mal-adjustment between the new system and the realization of grassroots governance is also increasingly prominent (Chan and Gao, 2008), which makes grassroots cadres adopt the coping strategies of “seeming to be busy” and “being busy but not moving ahead” when facing many uncertainties.

On the one hand, under the backdrop of administering the Party in a comprehensive and strict manner, the supervision mechanism of the Party and the state has been operated efficiently, and the political environment has been significantly improved. However, it has also brought about the problem of strengthened negative incentives and insufficient positive incentives. In the process of implementing accountability system, some localities have seen accountability chaos such as simplification, emotionalization and randomness, which has destroyed the “trust game” balance of the original incentive mechanism (Duan and Chen, 2021). Under the circumstance that the incentive mechanism and protection mechanism are not clear, some cadres have the motivation to give up the “administrative discretion” on their own initiative and adopt the strategy of shifting from “taking responsibility” actively to “seeming to be busy” passively to avoid direct or potential accountability risks.

However, after entering a new era of socialism with Chinese characteristics, the central work of governments is increasingly arranged in the form of fuzzy tasks, and a fuzzy governance model has been formed to a certain extent, which can mobilize the subjective initiative and enthusiasm of grassroots cadres. However, the institutional gap brought by fuzzy governance will aggravate the uncertainty of task fulfillment of grassroots cadres (Wilkins, 2002; Davenport and Leitch, 2005). Moreover, due to the imperfect access system for the tasks to be deployed to the grassroots, in the real scene of power and responsibility hanging upside down, grassroots cadres have to deal with the indicators and tasks assigned by their superiors, and owning to limited time and energy, it is inevitable to alienate the “invalid busyness” behaviors of grassroots cadres.

### Technological governance paradox: Actor-technology

Huang (1981) emphasized that the lack of “mathematical management” was the reason for the ineffectiveness of traditional Chinese governance. Today, technological means are no longer the main obstacle restricting the improvement of governance capacity and have been widely used in national governance and social governance. Technological governance expands the scope of “mathematical management,” alleviates the problem of “information asymmetry,” and facilitates to improve the efficiency of governance. However, excessive reliance on technological governance may lead to counterproductive effects.

On the one hand, technological governance has standardized the operation of power through institutionalized administrative procedures and fine technologies and promoted the transformation of cadre assessment from “result-oriented” to “process and result oriented.” However, excessive “trace management” has evolved into “trace doctrine” in the whole process of decision-making, execution, and assessment. In recent years, the grassroots governance has been inundated with traces, such as taking photos, punching cards, GPS, etc., resulting in wasting a great deal of manpower, material and financial resources, and idling the system (Tang, 2021). This not only distorts the concept of political achievements of grassroots cadres, but also leaves the public with a stereotype of bureaucratic style.

Technological governance tends to strengthen top-down control and “Technology Leviathan,” inducing grassroots cadres to transfer their discretion to “layer upon layer upward” and “algorithmic bureaucrats,” and “abstaining from power to avoid accountability” becomes the optimal solution for rational individuals. In addition, it is undeniable that technological means play a positive role in improving government performance and promoting social welfare. However, in recent years, it is also a fact that the grassroots pay attention to technological innovation while neglecting institutional mechanism innovation. Accordingly, the technology replicates the institution in the virtual space and strengthens the hierarchical relationship, resulting in an increase in the responsibilities and matters of the grassroots government (Mounier-Kuhn, 1994), and makes grassroots cadres fall into the paradox of technological governance.

## Correction mechanism for “invalid busyness” in grassroots cadres

**Figure 3:**
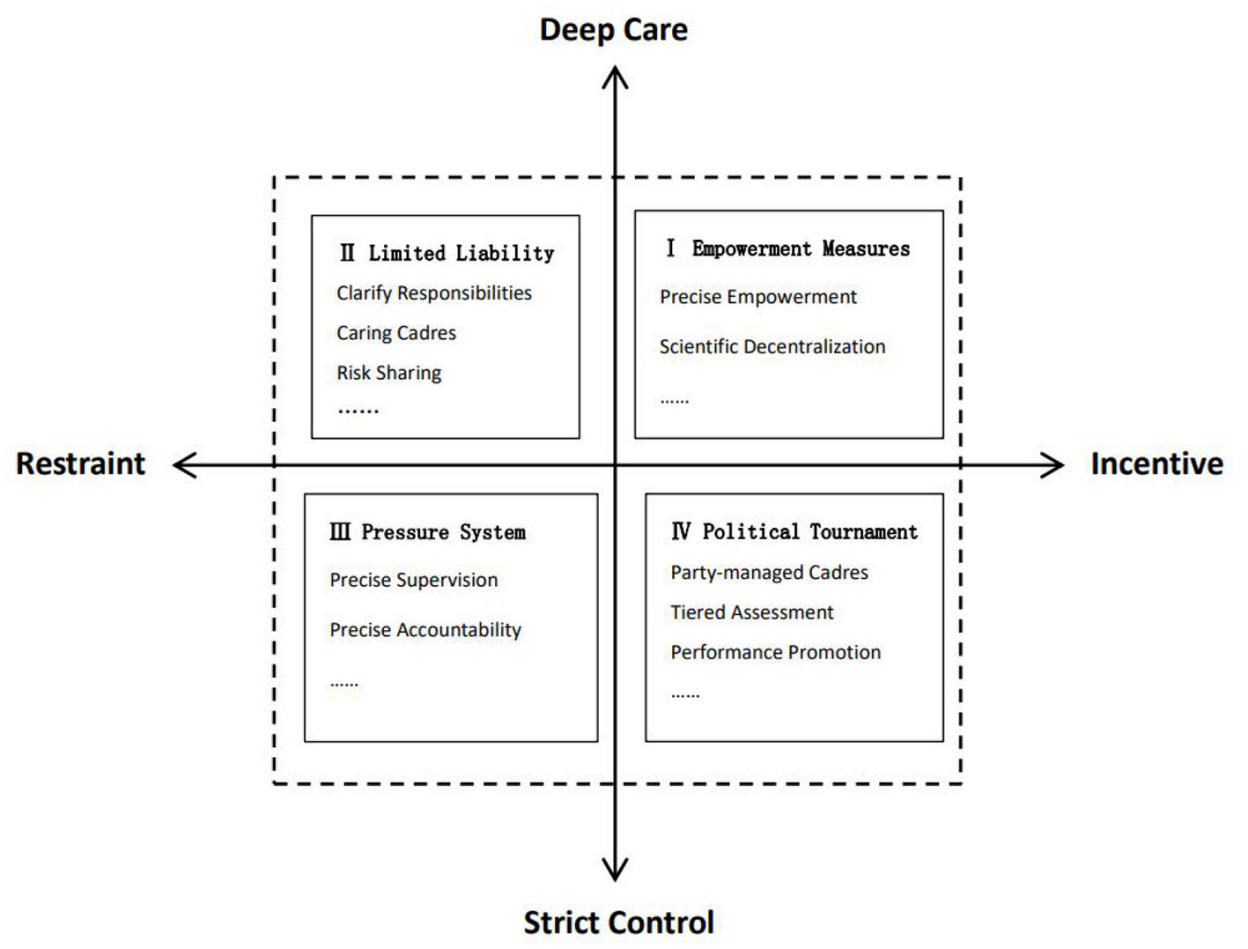
Correction mechanism of “invalid busyness” in grassroots cadres.

The grassroots are the “last kilometer” of national governance and the “first kilometer” of social governance. The level and efficiency of grassroots governance have a bearing on the overall development of the Party and the state. In the face of the current “invalid busyness” behaviors of some grassroots cadres, it is urgent to respond and correct them from theoretical and practical perspectives in a bid to better transform the institutional advantages into governance efficiency. According to the externalization and formation mechanism of the “invalid busyness” behaviors of grassroots cadres, they can be corrected from four dimensions including “incentive, restraint, deep love and strict control” (as shown in Figure 3).

### Strict control—restraint: Pressure system

The pressure system refers to the management mode of quantitative task decomposition and the materialized evaluation system adopted by the political organizations in order to achieve economic catch-up and complete various indicator tasks assigned by the superiors (Gao, 2017). The corrective effect of the pressure system on the “invalid busyness” behaviors of grassroots cadres is mainly reflected in the use of precise supervision and precise accountability and other negative incentive mechanisms to identify those who are busy for nothing and ensure that the cadres take responsibility. Undoubtedly, for China in the period of economic transformation and social transition, exerting appropriate political pressure on the cadres through the precise supervision and accountability is not only an important measure to promote the decision-making and attention distribution of leading cadres, but also a crucial guarantee for the realization of responsible politics and responsible government.

On the one hand, the problem of “principal-agent” relationship between the central and local governments caused by the super large bureaucratic system has prolonged the organizational hierarchy chain, resulting in huge organizational efficiency loss and the stability of the central authority (Zhou, 2011). Therefore, in order to solve the internal contradiction between the unified system and effective governance, it is necessary to supervise and urge all power units to firmly establish the concepts of political achievements and power with a focus on responsibility and the people through the precise supervision mechanism to prevent honest cadres who dare to assume responsibilities from suffering losses, increase the probability that invalid busyness and idle behaviors are discovered and increase their pressure in order to achieve a reasonable balance between constraints and efficiency of grassroots governance.

Under the influence of such factors as formalization, simplification, randomness and selection of accountability, the grassroots cadres have a negative blame avoidance mentality (Weaver, 1986) from seeking “maximum political performance” in the past to seeking “minimum risk” at present, which will not only delay the development opportunity of the Party and the country but also erode the foundation of the Party's ruling and rejuvenating the country. It is urgent to start with the precise accountability work, carry out fine management on the taking-charge behaviors and work style of the cadres, and the cadres who are mediocre, lazy, and idle in governance shall be accurately identified and dealt with by means of transfer and dismissal to achieve the deterrent effect of “accountability, deterrence and education.”

### Strict control-incentive: Political tournament

Political tournament refers to that an individual's reward in a tiered tournament depends on his relative performance with respect to others (Lazear and Rosen, 1981). When applied to the political context in China, it refers to the promotion game process in which cadres develop local economy to seek job changes. The correction effect of the political tournament on the “invalid busyness” behaviors of grassroots cadres is reflected in the identification of doers through the positive incentive mechanisms such as placing cadres under Party supervision, hierarchical assessment and performance and promotion to drive the grassroots cadres to “take actions actively and vigorously.”

First, the Party supervises the cadres. The principle of placing cadres under Party supervision is not only an important lever to regulate the relationship between the upper and lower levels of government, but also an important prerequisite for an effective political tournament. This requires the cadre management department to select and train cadres according to the strategic layout and development tasks of the Party and the country, train cadres precisely and meticulously, care for cadres sincerely, manage cadres strictly and practically, and conduct strict management and training for some grassroots cadres with knowledge gaps, experience blind areas, and lack of ability to help them develop their abilities and qualities that are compatible with the mission of the times.

Second, tiered assessment. The change of the theme of governance affects the content orientation of the performance appraisal of Party and government cadres, which requires that the role and function of grassroots cadres in national governance be clarified, the special functions and values of grassroots cadres in implementing national policies and directly responding to the demands of the people be brought into play, the grassroots cadres be liberated from some futile and ineffective “idle” matters to give full play to the role of “fighting fortress” in resolving contradictions and risks at the grassroots level.

Third, performance promotion. Due to limited posts and promotion space for cadres, promotion incentive is the most direct incentive measure with the greatest impact. It plays a dual role of “wind vane” and “baton” for the performance of cadres. This requires that under the system and mechanism of placing the cadres under the Party's supervision, the departments in charge of personnel appointment and removal should establish a correct orientation for the employment of cadres so that “taking-charge” grassroots cadres can also “have a position,” and cadres who are busy working with effective and conducive results can be promoted and placed in key positions. In addition, to enhance the professional attraction of grassroots work and the enthusiasm of cadres to work diligently, it is also necessary to form an all-round incentive synergy through such incentive mechanisms as material, political, spiritual and emotional incentives to encourage the cadres to fulfill the tasks of the Party and the state in a down-to-earth manner and solve the problems concerned by the people.

### Incentive—deep care: Empowerment measures

The mechanism of empowerment and delegating powers to lower levels refers to endowing the working entities with power and capacity to stimulate their working potential (Quinn et al., 2015). The corrective effect of such a mechanism on the “invalid busyness” behaviors of grassroots cadres is reflected in “empowering” the cadres and “delegating powers” to the grassroots government so that the cadres “are courageous to undertake and fulfill tasks efficiently.” On the one hand, improving the empowerment mechanism for cadres to perform their duties is a dual need of national and social development, as well as a vital guarantee for grassroots cadres to be busy working in an effective and beneficial manner. First, it is necessary to strengthen the cadres' ideal and belief education, carry out effective political building for grassroots cadres, enhance their sense of professional significance and job responsibility, and strengthen their subjective initiative and public service motivation to stimulate their endogenous motivation to take the initiative.

Second, it is necessary to strengthen the cadres' professional skills training, precisely empower grassroots cadres for the efficient implementation of the work, enable cadres to “do through learning” and “learn by doing,” and resolve the “invalid busyness” problems that “old knowledge” does not work and “new knowledge” is not applied appropriately; Third, it is necessary to enrich the practical training and work experience of grassroots cadres, strengthen their ability to deal with complex problems and urgent, difficult, dangerous and heavy tasks to avoid letting grassroots cadres fall into the “invalid busyness” state.

On the other hand, “unable to hold the power” yet “having to hold it” is an important reason leading to the “invalid busyness” behaviors of grassroots cadres. Though several reforms of streamlining and delegating powers to lower levels have been carried out, the grassroots governments become more averse to such reforms because the corresponding resources are not allocated to the grassroots concurrently with the reforms which instead become a legitimate reason for “responsibility shifting,” that is, the superiors delegate the troublesome power to the grassroots (Gao, 2009). It can be seen that in order to liberate the grassroots cadres from the “invalid busyness” matters, the designers of the institution and mechanism need to promote investigation and research, scientifically evaluate the matching between the grassroots power and responsibility to ensure the power is equipped with supporting measures such as manpower, finance and materials in the process of delegating powers to lower levels so that the grassroots cadres can be busy working without aversion and the ordinary people can have more sense of gain.

### Deep care—restraint: Limited liability

The limited liability mechanism refers to the sharing of responsibilities between the upper and lower levels and the change of “unlimited liability” into “limited liability.” The corrective effect of the limited liability mechanism on the “invalid busyness” behaviors of grassroots cadres mainly includes three main links: clarifying responsibility in advance, caring in the process, and sharing risks after the event, with an aim to relieve the cadres of their worries. First, a “responsibility list” should be prepared in advance to form a risk sharing commitment mechanism. In the process of transferring governance focus to a lower level and carrying out reforms to streamline administration, delegate powers and improve management and service, the preparation of “responsibility list” is an important mechanism for clarifying responsibilities in advance, and is also a key criterion for regulating the performance of duties of grassroots cadres. It is conducive to preventing the superiors from transferring temporary and urgent tasks to the grassroots in the name of “streamlining administration and delegating power.” A scientific and reasonable responsibility list needs to be designed and adjusted according to the actual situation of grassroots work and the real demands of the people based on sufficient investigation and research. Only in this way can grassroots cadres be busy but not in vain, and busy but effective.

Second, the protection mechanism for cadres in the process of the event should be established to ensure timely verification and clarification, as well as organizational support and care. As the reform enters the deep-water area, more courage and resolve are needed because the reform may easily move the “cheese” of others and is likely to be repelled by the counterparts, resulting in the negative effect of “bad currency expelling good currency.” In this regard, it is necessary to establish a rapid inspection and handling mechanism and a clarification and care mechanism to support cadres who are bold to assume responsibility, take actions in a down-to-earth manner and do not seek personal interests and to cultivate a sense of belonging and trust in the organization.

Third, the post-event liability sharing mechanism should be established and the system design of fault tolerance and error correction should be improved. As the dividends of reform and innovation subside and the pressure of accountability increases geometrically, many grassroots cadres are “invalid busyness” because they are afraid of making mistakes, and there even exists the dishwashing effect of “making more mistakes if doing more, making fewer mistakes if doing less” within the organization. In order to encourage and protect the cadres who dare to do things and work hard, it is necessary to establish and improve the fault tolerance and correction mechanism so that the assessment, appraisal and other measures that meet the fault tolerance and exemption conditions will not be affected and increase the endogenous impetus to encourage the grassroots cadres to be willing to be busy working in a real and effective manner.

## Conclusion and discussion

The “busyness” of grassroots cadres is a manifestation of hard work and the basis for success. There is nothing wrong with busyness. As the focus of social governance moves downward to the grassroots level, the tasks and responsibilities undertaken by grassroots cadres also increase. Busyness has become the normal state of grassroots work. However, busyness without success and busyness without benefit means fruitless busyness, empty busyness, and blind busyness.

At present, the ”invalid busyness“ behavior of some grassroots cadres will not only consume a large number of public resources and dampen the working enthusiasm of cadres, but also endanger the relationship between the Party and the ordinary people, affect the implementation of policies and work, and become a “stumbling block” for the modernization of the grassroots governance system and governance capacity. In view of this, on the basis of existing theoretical research and practical materials, this study analyzes eight typical types of “invalid busyness” behaviors of grassroots cadres, and explores the generation mechanism of “invalid busyness” behaviors under the theoretical framework of “environment, organization, institution, technology, and political man;” Moreover, it puts forward correction measures from four-dimensional logic including “incentive, restraint, deep love, and strict control,” and expands the research content of grassroots governance and reflects the realistic concern about the plight of grassroots governance to a certain extent.

This study finds that the breeding and spread of “invalid busyness” behaviors of some grassroots cadres are affected by multiple factors such as the paradoxes of risk society, territorial management, incentive intensity and technological governance. It is urgent to take measures such as negative incentives, positive incentives, empowerment, and limited responsibilities to stimulate the endogenous motivation of grassroots cadres to take solid actions and promote the high-quality development of grassroots governance. The taking-charge behaviors of grassroots cadres are complex and diverse. The active or passive “invalid busyness” behaviors of some grassroots cadres exist in various links and fields of grassroots governance. However, performing duties and assuming responsibilities in an earnest manner is still the mainstream among grassroots cadres. The crux of the occurrence should be grasped and properly adjusted and repaired, accordingly, grassroots governance will develop and advance in the correct direction toward better results.

### Theoretical implications

It is of great theoretical significance to systematically analyze the “invalid busyness” behavior of grassroots cadres, which is a common phenomenon in the process of grassroots governance and policy implementation. The research contributions of this paper are mainly in the following three aspects.

First, this paper systematically depicts the “manifestation-causes-cure” of grassroots cadres' “invalid busyness” behavior from a microscopic individual perspective, which enriches the theoretical study of bureaucratic behavior. In fact, the “invalid busyness” behavior of grassroots cadres is influenced by a variety of factors, but previous studies often analyze it from a single perspective, lacking a holistic framework to clarify the performance behavior of grassroots cadres (Sminth, 2010; Chen, 2020; Yang and Li, 2020). The analytical framework and perspective of this study expand the explanatory power of existing studies on the performance behavior of grass-roots cadres.

Second, for a long time, few scholars have studied grassroots cadres in their inferior position, but with the introduction of Lipsky's (1980) “street bureaucracy” theory, the performance behavior of grassroots cadres has become a hot issue in academic research. This paper extracts eight typical manifestations of grassroots cadres' “invalid busyness” behavior, which enriches the theoretical research and empirical evidence of “street bureaucrats” in light of the new changes in their performance behavior.

Finally, the “pressure-response” model is a classic theory to explain the performance behavior of public officials, in which “response” is usually divided into top-down political responsiveness and bottom-up social responsiveness (Park and Han, 2018). In this paper, we found that under multiple task pressure situations, public officials selectively respond to top-down political pressure and intentionally or unintentionally avoid bottom-up social demand pressure, which is consistent with previous studies (Yang and Yu, 2012; Duan, 2021; Tu and Gong, 2021).

### Managerial implications

Since the tax and fee reform, the grassroots authorities have been in a relatively loose “hollowed” relationship with farmers for a long time (Sminth, 2010). However, as grassroots governance gradually enters the transition period of “bringing the state back,” there will be a lot of hidden and explicit contradictions, old barriers and new demands, which need the attention of practical departments and governance subjects.

On the one hand, good grassroots governance is our common ideal, but it should not be overly idealized. In the face of the ever-changing governance environment, we should adopt an inclusive attitude, regard the spiral development of grassroots governance as a gradual process, allow grassroots cadres to have room for flexible governance and flexible response, and summarize and correct the deviations of grassroots governance in a timely manner, so as to guarantee the stability of grassroots governance and the long-term stability of national governance.

On the other hand, although some grassroots cadres have been “invalid busyness” in the process of grassroots governance, which is different from the requirement of performing their duties and responsibilities, we should not be too pessimistic because “common sense tells us that even the most spectacular successes include some minor failures, or only a few (McConnell, 2011), which is in line with this paper's intention to emphasize ”some“ grassroots officials' “invalid busyness” behavior, but should also attract the attention and vigilance of relevant departments to prevent ”minor illnesses“ from becoming ”major disasters, to free grassroots cadres from all kinds of “invalid busyness” affairs, and to give them more time and energy to do practical work for the people, to do a good job in serving the masses, and to truly bring into play the actual effectiveness of grassroots governance.

## Data availability statement

The original contributions presented in the study are included in the article/supplementary material, further inquiries can be directed to the corresponding author.

## Author contributions

CD contributed to the initial idea, framework, first draft, and the supervision of the total work in the process. YZe and YL contributed to supporting all the work of the CD. YZh contributed to develop the research framework. All authors contributed to the article and approved the submitted version.

## Funding

This research was supported by the National Social Science Fund Youth Project a study on the - Invalid Busyness phenomenon in a few grassroots cadres and its correction mechanisms (No. 21CZZ036).

## Conflict of interest

YL was employed by company Shenzhen Airport. The remaining authors declare that the research was conducted in the absence of any commercial or financial relationships that could be construed as a potential conflict of interest.

## Publisher's note

All claims expressed in this article are solely those of the authors and do not necessarily represent those of their affiliated organizations, or those of the publisher, the editors and the reviewers. Any product that may be evaluated in this article, or claim that may be made by its manufacturer, is not guaranteed or endorsed by the publisher.
